# Factors Affecting User’s Behavioral Intention and Use of a Mobile-Phone-Delivered Cognitive Behavioral Therapy for Insomnia: A Small-Scale UTAUT Analysis

**DOI:** 10.1007/s10916-021-01785-w

**Published:** 2021-11-12

**Authors:** Siska Fitrianie, Corine Horsch, Robbert Jan Beun, Fiemke Griffioen-Both, Willem-Paul Brinkman

**Affiliations:** 1 Delft University of Technology, Delft, the Netherlands; 2 Utrecht University, Utrecht, the Netherlands

**Keywords:** Acceptance research, UTAUT2, Insomnia, Smartphone app, Cognitive behavioral therapy, eHealth, Partial least squares-structural equation modeling

## Abstract

A mobile app could be a powerful medium for providing individual support for cognitive behavioral therapy (CBT), as well as facilitating therapy adherence. Little is known about factors that may explain the acceptance and uptake of such applications. This study, therefore, examines factors from an extended version of the Unified Theory of Acceptance and Use of Technology (UTAUT2) model to explain variation between people’s behavioral intention to use a CBT for insomnia (CBT-I) app and their use-behavior. The model includes eight aspects of behavioral intention: performance expectancy, effort expectancy, social influence, self-efficacy, trust, hedonic motivation, anxiety, and facilitating conditions, and investigates further the influence of the behavioral intention and facilitating conditions on app-usage behavior. Data were gathered from a field trial involving people (*n* = 89) with relatively mild insomnia using a CBT-I app. The analysis applied the Partial Least Squares-Structural Equation Modeling method. The results found that performance expectancy, effort expectancy, social influence, self-efficacy, trust, and facilitating conditions all explained part of the variation in behavioral intention, but not beyond the explanation provided by hedonic motivation, which accounted for *R*^2^ = 0.61. Both behavioral intention and facilitating conditions could explain the use-behavior (*R*^2^ = 0.32). We anticipate that the findings will help researchers and developers to focus on: (1) users’ positive feelings about the app as this was an indicator of their acceptance of the mobile app and usage; and (2) the availability of resources and support as this also correlated with the technology use.

## Introduction

More than 300,000 unique healthcare available in major app stores and much work has addressed the feasibility and efficacy of these apps [1]. Despite promising results, the impacts of a health app could be lost if users reject using the app. Hence the question: what are factors that inhibit or enhance the actual use of these apps? Although Unified Theory of Acceptance and Use of Technology (UTAUT) [2–4] shows factors influencing technology acceptance in various domains, less is known about these factors in the context of health apps. Therefore, we report a case study set the insomnia treatment domain.

Insomnia is a sleep disorder where people have trouble with falling asleep or staying asleep. About 10 − 15% of adults report symptoms of insomnia associated with daytime consequences, and it is estimated that 6 − 10% of adults meet the criteria for an insomnia disorder [5]. Currently, Cognitive Behavior Therapy (CBT) is widely recognized as an effective non-pharmacological treatment for insomnia. Van Straten et al. [6] reported that a CBT for insomnia (CBT-I), either its components or the full package, was effective for treating insomnia.

CBT is a short-term psychotherapy that helps patients change self-limiting beliefs, develop new behaviors, and manage emotions. CBT-I techniques include relaxation, sleep restriction, stimulus control, cognitive therapy, and sleep hygiene. Relaxation exercises help the person to relax and to attain a state of calmness. Sleep restriction involves controlling time in bed based on a person’s sleep efficiency to restore the homeostatic drive to sleep. On the other hand, stimulus control aims at restoring the person’s positive association of the bed and bedroom with sleep. Cognitive therapy aims at changing dysfunctional beliefs and attitudes, whereas, sleep hygiene education aims to increase awareness of the behaviors and environmental factors that promote sleep.

Internet-delivered CBT-I has been developed to improve the availability of personal therapists by promoting adherence at a low cost and providing both long-distance communication and tailored interaction. Ritterband et al. [7] showed large improvement effects as a result of fully automated CBT-I treatment. Given advancements in mobile technology, an effective-CBT-I app could offer an opportunity to enhance the advantages and coverage provided by other formats of CBT-I, enabling real-time and personalized monitoring, assessment, and interventions [8, 9]. In particular, our developed SleepCare app was a stand-alone mobile phone delivered CBT-I and packaged with a sleep diary, a conversation module, a relaxation exercise, a sleep-restriction exercise, and sleep hygiene and education [8–13]. Horsch et al. [13] reported moderate significant effects of using this app according to insomnia severity index (ISI *d* = -0.66) and sleep efficiency (*d* = 0.71), which were also maintained at a three-month follow-up.

To the best of our knowledge, no studies have evaluated the acceptance and use of CBT-I apps. To understand factors that may explain the adoption of a CBT-I app, this paper presents an analysis of users’ behavioral intentions to use the app and their use-behavior, and underlying factors that could explain variation between users on these factors. The analysis was based on data obtained as part of a randomized controlled trial. It focused only on the experimental condition in which people with insomnia used the SleepCare app for seven weeks (*n* = 89). The Partial Least Square Structural Equation Modeling (PLS-SEM) method was chosen for this study due to its ability to handle heterogeneous data with a small sample size [14].

## Research model

**Fig. 1:**
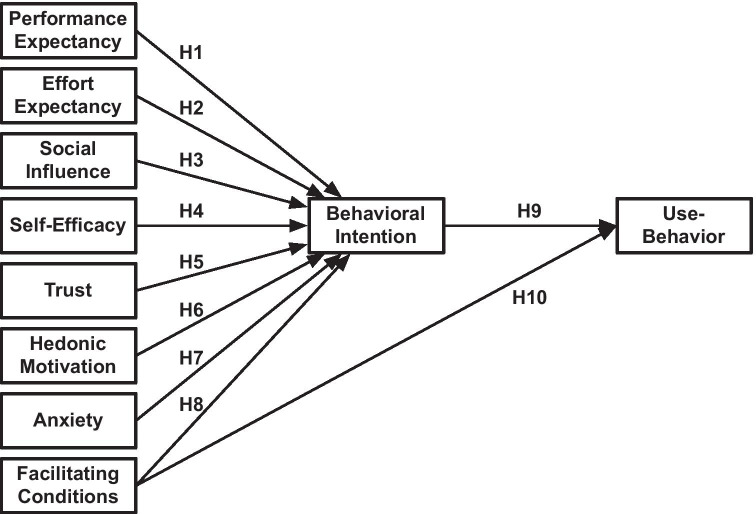
Research model

With CBT-I, individuals engage deliberately in activities that are expected to reduce their insomnia symptoms. They make conscious attempts to affect behavior that is not under volitional control, such as falling asleep or waking up. Their behaviors are shaped by beliefs towards the changed behavior and the support tools. This process can be depicted as a model of factors that determine the acceptance of app-based CBT-I and the related behavior (Fig. 1).

The research model was developed based on the extended model of the Unified Theory of Acceptance and Use of Technology (UTAUT2) [3], i.e. a single theory combining many theories often used to understand technology use, which includes: the Theory of Reasoned Action, the Theory of Planned Behavior, Self-Determination Theory, Innovation Diffusion Theory, the Motivation Model, Social Cognitive Theory, the Model of Personal Computer Utilization, and the Technology Acceptance Model. Since its introduction, the UTAUT model [2] has been applied in multiple domains [4]. The meta-analysis using the original UTAUT model yielded medium to large associations between the UTAUT determinants and behavioral intention [15]. This model has often been extended beyond its original remit to encompass determining factors of behavioral intention, namely performance expectancy, effort expectancy, and social influence [3, 4]. In UTAUT2, Venkatesh et al. extended the model to include facilitating conditions, hedonic motivation, price value, and habit as determinants of behavior intention [3]. Table 1 gives an overview of the adaptation of these determinants for this study.

**Table 1:** Factor affecting behavioral intention and use-behavior

Constructs	Theoretical Definition	Definition in the CBT-I app domain
Performance Expectancy	The degree to which an individual believes that using the system will help him/her to attain gains in job performance [2]	The user’s expectation that the app can help them perform their therapy
Effort Expectancy	The degree of ease associated with the use of the system [2]	The user’s beliefs about the ease of using the app
Social Influence	The degree to which an individual perceives that important others believe he/she should use the new system [2]	The user’s perception of the social pressures to use the app
Self- Efficacy	An individual’s beliefs about his/her capabilities to produce designated levels of performance that exercise influence over events that affect their lives [16]	The degree to which the user determines themselves capable of using the app for the treatment of insomnia
Trust	How trustworthy one finds a system concerning its capability, reliability, and credibility [17]	The user’s feeling of security and willingness to depend on the app for the treatment of insomnia
Hedonic Motivation	An individual’s liking for a particular behavior [18]; the fun or pleasure derived from using a technology [3]	The user’s positive feeling about using the app
Anxiety	Evoking anxious or emotional reactions when it comes to performing a behavior [2]	The user’s lack of anxiety about using the app
Facilitating Conditions	The degree to which an individual believes that an organizational and technical infrastructure exists to support the use of the system [2, 3]	The user’s perceptions of the resources and support available to use the app
Behavioral Intention	The degree to which an individual formulates conscious plans to perform a behavior [2, 3]	The user’s intent to use the app, follow its advice, and perform the advised CBT-I exercises
Use-Behavior		The user’s behavior involves doing exercises and engaging with the app

According to the UTAUT2 model, people’s behavioral intention is a key mediating determinant for behavior under volitional control. Therefore, the factors considered influencing the intention in this study are limited to those factors that people can explore consciously, and hence people can be queried about them. These factors are expected to shape people’s intentions to engage in specific behavior and are therefore regarded as predictors for intentions—or in this case, the decision to use a CBT-I app. While including hedonic motivation, i.e. the user’s positive feeling about using the app, this study excluded two factors of UTAUT2: price value and habit, because the participants of the study did not have to buy the app and the seven-week limitation of interacting with the app was not regarded long enough for these participants to form a habit. Instead, the study considers three additional determinants because of previous findings. They are: (1) *self-efficacy*, as it has a strong relationship with users’ intention to use healthcare technologies [19]; (2) *trust*, as it influences users’ intention to use health information systems [20]; and (4) *anxiety*, as it was a significant predictor for users’ intention to use information technologies [21, 22].

For technology use, however, individuals’ perceptions of the available resources and support to help them use the technology are also important [2, 3]. Hence, UTAUT2 includes facilitating conditions as a second determinant [3]. The meta-analysis of Khechine et al. [15] reported medium associations between the UTAUT determinants, i.e. facilitating conditions and behavioral intention, and use-behavior.

In the CBT-I delivered by the SleepCare app, user behaviors consist of exercise behaviors (cf. main activities) and app-related activities (cf. supporting activities) [8, 9]. The former refers to actions that are usually related to the advised exercises, e.g. sleep restriction and relaxation exercise; and the latter is related to actions which the users engage in with the app, e.g. journaling using the sleep diary, and having consultations or dialogues with the app. Therefore, this study had defined one construct to capture these user behaviors: *use-behavior*.

## Method

To identify significant influence within the relationships included in the model (Fig. 1), the present study analyzed 10 hypotheses (Table 2).

**Table 2:** List of hypotheses tested in the study

H	Description
Factors affecting Behavioral Intention	
H_1_	Users’ performance expectancy positively correlates with their behavioral intention to use the app
H_2_	Users’ effort expectancy (ease of use) positively correlates with their behavioral intention to use the app
H_3_	Users’ social influence positively correlates with their behavioral intention to use the app
H_4_	Users’ self-efficacy positively correlates with their behavioral intention to use the app
H_5_	Users’ trust positively correlates with their behavioral intention to use the app
H_6_	Users’ hedonic motivation positively correlates with their behavioral intention to use the app
H_7_	Users’ lack of anxiety positively correlates with their behavioral intention to use the app
H_8_	The app’s facilitating conditions positively correlates with the user’s behavioral intention to use the app
Factors affecting Use-Behavior	
H_9_	The user’s behavioral intention positively correlates with their use-behavior
H_10_	The app’s facilitating conditions positively correlates with the user’s use-behavior

The data was collected as part of a randomized controlled trial. The goal of the trial was to demonstrate the app’s efficacy in a sample of patients with relatively mild insomnia to test the proof of principle before investigating it in a more severely affected population. Although the trial had both app- and waitlist-conditions, only data collected from participants who received CBT-I treatment was examined in this study. In this section, the demographics of participants, a description of the app, the measurement instruments, the procedure of the study, and the data analysis are presented.

### Participant

**Fig. 2:**
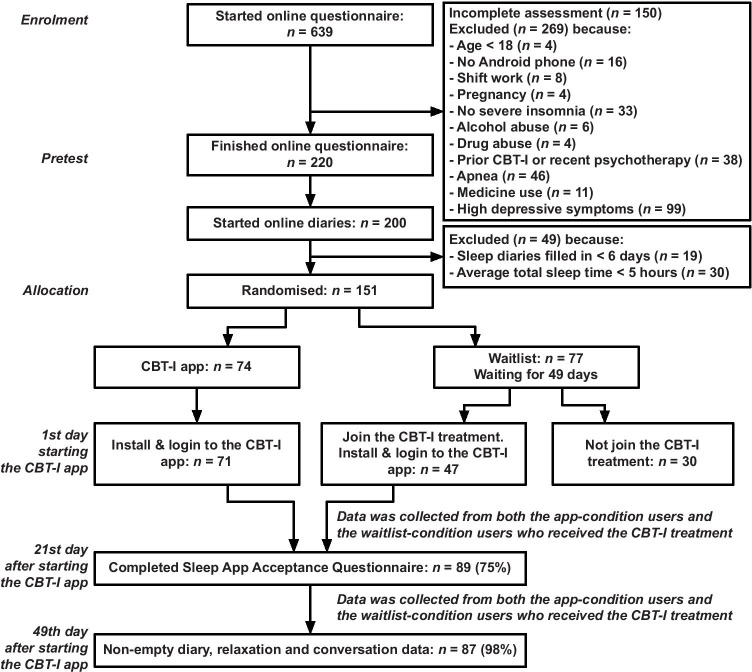
CONSORT flow diagram of recruitment, reasons for exclusion, experimental compliance, modified version of [13]

The participants in this study were recruited from August 15 to October 21, 2015, via websites, social media, online advertisements, flyers, and a press release. 151 participants out of an initial group of 639 voluntarily recruited individuals were included based on the inclusion and exclusion criteria (Fig. 2, [23]). The *inclusion criteria* [13] were: (1) difficulty with initiating or maintaining sleep for at least 30 min a night, for at least 3 nights a week, for at least 3 months, causing clinically significant distress or impairment in daily functioning, under the criteria for the Diagnostic and Statistical Manual of Mental Disorders (DSM-5) diagnosis of insomnia [24]; (2) stable medication use; (3) being eighteen years or older; and (4) having a valid email address, being connected to the internet, and in possession of an Android mobile phone (operating system version 4.1 or higher). The *exclusion criteria* were: (1) total sleep time < 5 h on average as reported in a 7-day sleep diary; (2) Insomnia Severity Index (ISI) score lower than 7 [25]; (3) earlier treatment with CBT-I; (4) started other psychotherapy in the last six months; (5) self-reported diagnosis of schizophrenia or psychosis; (6) alcohol or marijuana abuse (more than three glasses of alcohol a day for at least 21 days a month or use marijuana more than once a week); (7) possible sleep apnea (determined with a subscale of the SLEEP-50 questionnaire [26], cut-off ≥ 15); (8) shift-work; (9) pregnancy or breastfeeding; or (10) symptoms of depression (determined with a subscale of the Centre for Epidemiological Studies Depression (CES-D) scale [27, 28], cut-off ≥ 27).

118 participants installed the app on their mobile devices, but only 89 participants filled in the tailor-made Sleep App Acceptance Questionnaire. Table 3 shows the demographic data of these participants.

**Table 3:** Demographic characteristics of participants in the Sleep App Acceptance Questionnaire based on [13]

Characteristics		Sample (*n* = 89)
Age in years, mean (SD)		42.0 (13.5)
Sex, *n* (%)		
	*Female*	52 (58.4)
	*Male*	37 (41.6)
Living together, *n* (%)		
	*Yes*	62 (69.7)
	*No*	27 (30.3)
Employee, *n* (%)		
	*Yes*	67 (75.3)
	*No*	22 (24.7)
Educational Level, *n* (%)		
	*Lower gen. secondary*	5 (5.6)
	*Higher gen. secondary*	9 (10.1)
	*Community College*	10 (11.2)
	*University*	65 (73.0)
Duration of insomnia, years, *n* (%)		
	< *1*	11 (12.4)
	*1–5*	35 (39.3)
	*5–10*	11 (12.4)
	> *10*	26 (29.2)
	*Unclear answer*	6 (6.7)
Due to a physical condition, *n* (%)		
	*Yes*	9 (10.1)
	*No*	80 (89.9)
Used sleep medication, *n* (%)		
	*Yes*	8 (9.0)
	*No*	81 (91.0)
Insomnia Severity Index, mean (SD)		
	*Pretest*	16.6 (3.0)

### System description

The SleepCare app [8, 9, 11–13] was based on previously published protocols (e.g. [29, 30]). It offered a seven-week CBT-I program, which consisted of six weeks of CBT-I exercises (as recommended by [6]) plus an additional one week for managing the participants’ adherence. The interface of the app was composed of a set of personalized conversations and specialized modules that formed a coherent structure of input and output facilities [9]. The conversations included introductions to the exercises, tailored advice for the individual user to do the exercises, appointments for consultations, shared decision-making about exercise-related assignments, evaluations of progress in the therapy, and feedback based on the current state of the user [11]. The app was fully automated.

The SleepCare app offered a sleep diary, a relaxation exercise, a sleep-restriction exercise, and sleep hygiene and sleep education. These exercises were in Dutch, tailored to the participants. The *sleep diary* was introduced on the first day of the therapy and could only be done for the current day. The *relaxation exercise* was introduced on the fourth day. The user was advised to do the relaxation once a day, but they could do it as often as they wanted [31]. After a week or when the condition that the user had filled out at least six sleep diaries and had an average sleep efficiency of less than 85%, the app introduced the *sleep-restriction* exercise. For this exercise, the app was equipped with an algorithm to calculate the ideal and maximum time in bed, creating boundaries for negotiation [13]. Finally, *sleep hygiene and sleep education* was presented as tips and facts that aimed for increasing the efficacy of the other exercises, in text format on a dedicated screen.

### Measurement

#### Questionnaire measures

A tailored questionnaire, i.e. Sleep App Acceptance Questionnaire, was developed to measure the following constructs: Performance Expectancy, Effort Expectancy, Social Influence, Self-Efficacy, Trust, Hedonic Motivation, Anxiety, Facilitating Conditions, and Behavioral Intention. Appendix A shows the list of items used in the questionnaire, translated into English. Each item was rated on a 7-point Likert scale (from 1 = strongly disagree to 7 = strongly agree) including a “not applicable” option which the participants could select if the item did not apply to them.

#### Behavioral measures

Recorded interaction data of the participants were processed to measure one use-behavior construct that consisted of three indicators: (1) the number of days the sleep diary was completed (SD-DO); (2) the number of completed conversations (CONV-DO) and (3) the number of days the participant did at least one relaxation exercise (RE-DO).

### Procedure

Participants gave online informed consent and filled in the questionnaire asking about the inclusion and exclusion criteria (including ISI) and demographics. The participants who met the criteria received a link to an online sleep diary by e-mail. For seven successive days, emails for the diary were sent at 6 a.m. and a reminder was sent at 10 a.m. Participants who reported an average total sleep time of more than five hours were included in the later stage of the study and randomly assigned to the app-condition or the waitlist-condition. The app-condition participants were allowed to install the app, while the waitlist-condition had to wait seven weeks before they were also allowed to enter the app-condition and install the app.

Three weeks after starting to use the app, all participants in the app-condition received another ISI questionnaire and the Sleep App Acceptance Questionnaire. At the same time, they received additional questionnaires to measure their dysfunctional beliefs about sleep and their motivation to use the app. At the end of seven weeks, all participants in the app-condition received a request to complete additional questionnaires, which also included the final ISI questionnaire. Again, they received additional questionnaires to measure their dysfunctional beliefs about sleep, anxiety, and depression symptoms. They were also asked to fill in a seven-day online diary to measure sleep variables such as sleep efficiency. After completing the online diary, the participants received a three-month follow-up questionnaire and another online diary assignment.

Note that the analyses of ISI, the dysfunctional beliefs about sleep, anxiety and depression symptoms, and the online diary assignments completed by the participants have been reported elsewhere [13].

### Data analysis

In this study, two types of PLS-SEM tests were conducted: (1) single-path tests that examined the relationships between two latent variables in the model separately, and (2) multiple-path tests that examined all the relationships in the model as a whole. The tests were analyzed using SmartPLS 3.0 [32]. Nonparametric bootstrapping-based 95% confidence intervals were computed and used to determine the significance of estimates. Data, scripts, and analysis files are online available [33].

Additionally, mediation analysis [34] was conducted to examine the mediation of the Behavioral Intention in the relationship between significant determinants (of Behavioral Intention) and Use-Behavior. Behavioral Intention was hypothesized to play a mediating role between variables early and later in the causal chain. For example, the Behavioral Intention construct clarifies how beliefs about expected performance influence the usage of the app.

#### Minimum sample size

Initial sample size estimation was primarily guided by the objective of comparing intervention and waiting list conditions of the trial. Fortunately, data collected in the intervention condition also abided to recommendations for PLS-SEM (Table 4), although not for the more traditional covariance-based SEM. The latter would require a recommended minimum sample size of 200 [35] as it pursues different objectives to this study, i.e. to test and confirm a prior theory. Instead, PLS-SEM with its explorative nature was used to examine relationships between the latent variables as hypothesized. The 89 samples collected falls within the range of minimum sample sizes recommended by various methods for PLS-SEM (Table 4), when pursuing a statistical power of 0.80 for detecting at least a medium-size effect of *R*^2^ (with 0.05 probability of error).

**Table 4:** Recommended minimum sample size for PLS-SEM

Method	Recommended Sample Size
10-times rules method [36]	80^a^
Minimum *R*^2^ method [14]	84
Gamma-exponential method [37]	99^b^
Inverse square root method [37]	85^b^

**Fig. 3:**
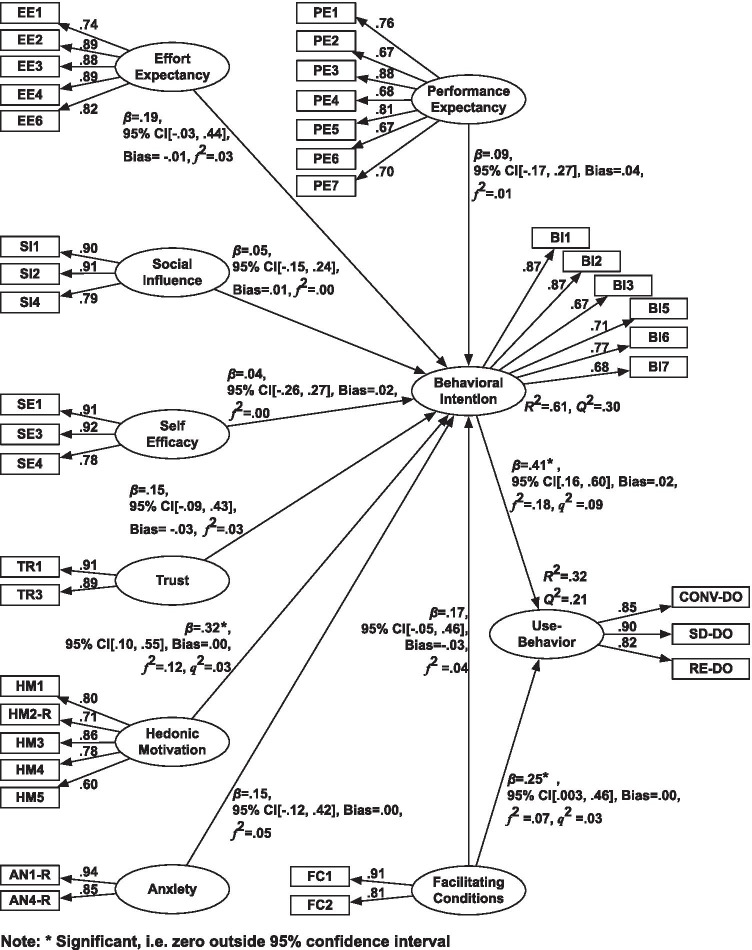
The bootstrapping results of the multiple-path analysis (*n* = 89)

The estimation was based on the results in Fig. 3

^a^8 independent variables in the outer model and

^b^the minimum significance of *β* = 0.25

#### Data characteristics

We considered an item in the questionnaire results as an outlier concerning its representativeness for the application domain if the number of participants who rated the item as ‘not applicable’ was more than Q3 + 1.5*IQR, with Q3 as the third quartile and IQR as the interquartile range of the ‘not applicable’ frequency across the questionnaire item measured. This led to the exclusion of items BI4, SI3, SE2, HM6, FC3, and FC4 from the analysis. For the remaining items, ‘not applicable’ was treated as a missing value. Only items BI1, BI2, BI3, and BI7 were answered by all participants. This resulted in 90 (2.3%) missing values with two participants out of 89 (5.6%) were missing all their behavioral data. The missing values were replaced using multivariate imputation based on a Bayesian linear regression method using the R version 3.3.3 mice package version 2.25 [38]. A detailed description of the measurement variables (*n* = 89) can be found in Appendix B.

#### Latent variable assessment

In this study, all variables were considered as reflective latent constructs and were assessed based on four criteria, as shown in Table 5. The assessment resulted in the removal of EE5-R, SI5-R, TR2-R, HM7-R, AN2-R, and AN3-R from the model (Appendix B).

**Table 5:** Assessment methods of reflective constructs

Assessment	Description	Criteria
Indicator Reliability	Describes the relationship among indicators being consistent with its construct	The outer loading of indicators > 0.50 is considered acceptable [39]
Internal Consistency Reliability	The inter-correlation of indicators from the same construct	Cronbach’s α > 0.70 is considered as modest [14]
Convergent Validity	The degree to which an indicator correlates with other indicators of the same construct	Average Variance Extracted (AVE) > 0.50 indicates that more than 50% of the variance from all indicators can be captured by the construct [36]
Discriminant Validity	Describes the distinctiveness of a construct from other constructs	No indicator has factor-loading on any other construct higher than the one it measures [40]

## Results

### Single path tests

Only Anxiety was not found to show a significant association with Behavioral Intention (Table 6). The other determinants, i.e. Performance Expectancy, Effort Expectancy, Social Influence, Self-Efficacy, Trust, Hedonic Motivation, and Facilitating Conditions, revealed associations with Behavioral Intention with medium (0.15 ≤ *f*^*2*^ < 0.35) to large effect sizes (*f*^*2*^ ≥ 0.35). Furthermore, the cross-validated redundancy values provided support for the models’ predictive relevance (*Q*^2^ > 0). Both Behavioral Intention and Facilitating Conditions were significant determinants for Use-Behavior. The association of Use-Behavior with Behavioral Intention could be classified as large, whereas with Facilitation Conditions was medium.

**Table 6:** The bootstrapping results of the single-path analyses (*n* = 89)

H	Construct	*R*^*2*^	*f*^*2*^	Predictor				*Q*^2^
				***β***	**95% CI**			
					**Low**	**High**	**Bias**	
Behavioral Intention as a dependent variable								
H_1_	Performance Expectancy	0.30	0.42	0.54*	0.39	0.66	0.03	0.14
H_2_	Effort Expectancy	0.31	0.45	0.56*	0.32	0.74	0.01	0.15
H_3_	Social Influence	0.22	0.27	0.46*	0.25	0.63	0.02	0.10
H_4_	Self-Efficacy	0.35	0.53	0.59*	0.33	0.76	–0.01	0.17
H_5_	Trust	0.28	0.40	0.53*	0.26	0.73	0.01	0.14
H_6_	Hedonic Motivation	0.45	0.82	0.67*	0.51	0.76	0.02	0.22
H_7_	Anxiety	0.12	0.13	0.34	–0.27	0.55	0.03	0.05
H_8_	Facilitating Conditions	0.25	0.34	0.50*	0.24	0.70	0.00	0.12
Use-Behavior as a dependent variable								
H_9_	Behavioral Intention	0.29	0.41	0.54*	0.36	0.67	0.02	0.19
H_10_	Facilitating Conditions	0.21	0.26	0.46*	0.28	0.60	0.01	0.13

*Significant, i.e. zero outside 95% confidence interval

### Multiple-path test

The multiple-path test result in Fig. 3 shows only Hedonic Motivation was a significant determinant for Behavioral Intention (H_6_). Furthermore, as in the single-path test, the analysis found that Behavioral Intention (H_9_) and Facilitating Conditions (H_10_) were both significant determinants for Use-Behavior. Only the association of Use-Behavior with Behavioral Intention could be classified as medium, while the other relationships revealed a small effect size (0.02 ≤ *f*^*2*^ < 0.15). Appendix B presents the results in a table format.

### Mediation analysis

**Fig. 4:**
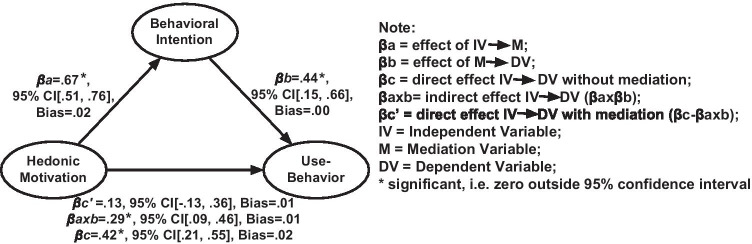
Effects of the relationship between Hedonic Motivation and Use-Behavior with mediation (*n* = 89)

The mediation analysis result showed that Behavioral Intention was fully mediating the relationship between Hedonic Motivation and Use-Behavior (Fig. 4). The mediation by Behavioral Intention on the relationships seems to be consistent with the research model. Appendix B presents the results in a table format.

## Discussion

**Fig. 5:**
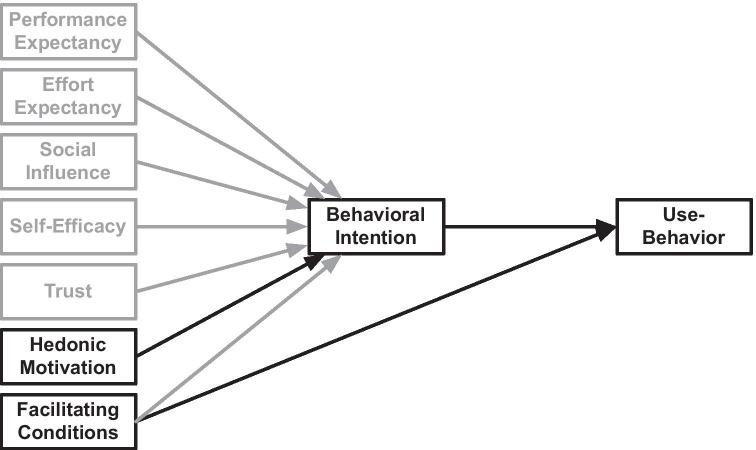
Conceptual model of the findings: the results of single-path analyses (grayed out) and multiple-path analysis (bold)

Fig. 5 presents the main findings of the study combining the single path analyses (grayed out) and the multi-path analysis (presented in bold). The findings of the single path analyses supported the viability of the model in assessing factors affecting behavioral intention to use the CBT-I app. The results showed that Performance Expectancy (H_1_), Effort Expectancy (H_2_), Social Influence (H_3_), Self-Efficacy (H_4_), Trust (H_5_), Hedonic Motivation (H_6_), and Facilitating Conditions (H_8_) could explain variation in the intention to use the app. However, the related multiple-path test revealed that the explanatory power of these individual factors did not go beyond the Hedonic Motivation factor, i.e. people’s positive feelings about the app, which accounted for 61% of the variance in Behavioral Intention between individuals. This result supported the inclusion of Hedonic Motivation in UTAUT2 [3]. Wang et al. [41] also found that the positive feelings about the system outperformed these traditional factors when explaining behavioral use among renal patients using a self-management support system.

In contrast to the results of Venkatesh et al. results [3], the findings of the multiple path analysis showed that Performance Expectancy, Effort Expectancy, Social Influence, and Facilitating Conditions did not significantly predict Behavioral Intention. One consideration could be that both this study and Wang et al. [41] used data obtained in the context of a trial. This is likely to suffer from a sampling bias. Participants who volunteer for this type of study may have prior beliefs that such an intervention would be beneficial for them, and confidence in their ability to use the technology to accomplish the tasks provided during the study. Consequently, this may have reduced the predictive power of the traditional UTAUT2 predictors. It seems that the hedonic motivation aspects are crucial when people have the freedom to choose. Comments made by the participants at the end of the study confirmed this. Some participants mentioned that they had fun and felt good using the app, as it helped them understand their sleep patterns. Others indicated that they needed more personalized features implemented in the app to help with their sleep problem. Without these aspects, as explained by Davis et al. [42], the expectations of the app’s performance and the social environment may influence people’s intention to use the technology for the first time. People may simply not know if they will like the app before they actually use it. In both this and Wang et al. [41] studies, however, determinants for behavioral data were collected when people had obtained some actual user experience.

Consistent with UTAUT2, Anxiety did not have a significant influence on people’s Behavioral Intention, and therefore no support was found for H_7_. One explanation could be inferred from the contextual factors, such as the substantial proportion of highly educated participants (i.e. about 73% were university graduates) and early middle-aged individuals, which together might indicate a demographics more at ease with using technology in this context.

The analysis also showed that Behavioral Intention (H_9_) and Facilitating Conditions (H_10_) were significantly associated with Use-Behavior, together accounting for 32% of the variance in people’s app interaction behavior. Both behavioral intention and facilitating conditions are important as they are predictors for people’s commitment to exercise behavior, in this case, journaling sleep diaries, having dialogues with the app, and doing the relaxation exercise. Furthermore, it revealed that Behavioral Intention fully mediated the impact of Hedonic Motivation on Use-Behavior. This finding was following other theories of behaviors which have long recognized Behavioral Intention as an important mediator in the relationship between behavior and other factors, such as attitudes or subjective and perceived behavioral control [3, 15, 17, 42].

In practice, the findings of this study suggest that the design and the implementation of a CBT app should take into account not only mobile phone-delivered CBT usability, social influences, and communicating potential benefits, but also the emotional experience of using the app. Some strategies for enhancing the emotional experience of app users were proposed by the participants at the end of the study. Very prominent were suggestions to automate diary entry and personalize the features developed in the app, such as the variation of exercises, music, and the voice used in the relaxation exercise, and also to personalize the conversation contents and the information displayed on the interface. Such improvements to the app were anticipated to increase the positive feeling of the users about the app. Other strategies have been proposed [8–10], which included usable and attractive design approaches for improving therapy adherence. Moreover, when offering therapeutic services on a mobile phone, it is also important to consider facilitating conditions, i.e. people’s perceived knowledge about using the app, and also the phone’s ability to run the app smoothly. Given the extensive variation in mobile phone models and different versions of operating systems in use [43], developers might be forced to find a balance between an attractive design and a solution with low demand for computational resources.

To fully appreciate the findings, understanding the study’s limitations is necessary. First, although to the best of our knowledge the data collected about mobile phone-delivered CBT-I was unique, the sample size was relatively small considering the number of factors included. A larger sample size would allow a confirmatory factor analysis and covariance-based structural equation modeling for objective estimation of the proposed model fit. Other limitations are related to the data collected in the study, which could put constraints on the generalizability of the findings and may cause a lack of consensus with other studies in the same context. The data collected by the questionnaires only provided snapshot information about beliefs three weeks after using the app. For example, the beliefs people held before downloading the app were not considered. Moreover, these questionnaires were tailor-made and adapted specifically to the purpose of the study. Another limitation is related to the representativeness of the Dutch sample for other countries, and the fact that a disproportionately large section of the sample consisted of highly educated participants. Furthermore, a substantial number of participants rated some of the questionnaire items, such as Behavioral Intention, Hedonic Motivation, Self-Efficacy, Social Influence, and Facilitating Conditions related to the mobile technological aspects, as not applicable. In addition, the assessment of latent variables led to the removal of six questionnaire items (observed indicators) that were negatively formulated. Reconsidering these items is therefore advisable for the future. Lastly, the technical issues that occurred during the trial made it impossible for some participants to continue to the next conversation. The occurrence of this problem was monitored and solved when needed. In these cases, a new conversation was manually planned in the database for these participants, and an email with instructions to update the app was sent to these participants.

## Conclusion

This study examined factors affecting users’ behavioral intention to use a cognitive behavioral therapy for insomnia delivered by a fully automated mobile app and the app-usage behavior. The study revealed that hedonic motivation (to use the app) was associated with people’s intention to use the app. As the behavioral intention was considered as an indication of the users’ acceptance, the results suggested that hedonic motivation modulated acceptance and in turn, together with facilitating conditions, affected their use-behavior with the app. These findings warrant more research into computerized persuasion strategies that support the hedonic motivation of behavioral change app users.

## Appendix A sleep-app acceptance questionnaire

***Note***. -R: answer needs to be reversed.

### Performance expectancy

**Table:**

PE1	The SleepCare app provides me with information about sleep
PE2	I feel relaxed assisting by the SleepCare app
PE3	The SleepCare app helps me to sleep better
PE4	The SleepCare app provides me clear insight into my sleep pattern
PE5	The SleepCare app provides me enough control over my own intervention
PE6	Using the SleepCare app, I can do the exercises independently and stay anonymous
PE7	The SleepCare app fits my personality

### Effort expectancy

**Table:**

EE1	The use of the SleepCare app gives me no ambiguities
EE2	The SleepCare app is easy to use
EE3	Learning to work with the SleepCare app is easy for me
EE4	Utilizing the app gives me little trouble
EE5-R	Using the SleepCare app is too complicated so I find it hard to understand
EE6	The app fits in with my daily life

### Social influence

**Table:**

SI1	I think that my friends would recommend I use the SleepCare app
SI2	I think that my family would think I should use the SleepCare app
SI3	I think that the people in my work environment would encourage me to use the SleepCare app
SI4	I think that other people with sleep problems would encourage me to use the SleepCare app
SI5-R	I think that my surrounding would think negatively of a person who uses the SleepCare app

### Self-efficacy

**Table:**

SE1	I can work well with the SleepCare app without help from others
SE2	I will be able to use the SleepCare app as long as nothing abnormal happens
SE3	I can work independently with the SleepCare app
SE4	I am sure that I’m using the SleepCare app in a good way

### Trust

**Table:**

TR1	I trust the information that the SleepCare app provides me with
TR2-R	I think the SleepCare app will put my privacy at risk
TR3	I am confident that the SleepCare app will work well

### Hedonic motivation

**Table:**

HM1	Using the SleepCare app is a good idea
HM2-R	I hate using the SleepCare app
HM3	I find that using the SleepCare app is fun
HM4	I find that using the SleepCare app is interesting
HM5	The SleepCare app gives me the feeling that my problem is taken seriously
HM6	The approach of the SleepCare app makes me feel safe
HM7-R	I feel apprehensive when using the SleepCare app

### Anxiety

**Table:**

AN1-R	I’m worried about using the app
AN2-R	I’m sometimes afraid of losing information if I press a wrong button
AN3-R	I’m afraid to make mistakes in the SleepCare app that I cannot turn them back
AN4-R	I feel somewhat intimidated by the SleepCare app

### Behavioral intention

**Table:**

BI1	I will definitely finish the training
BI2	I will definitely fill in the sleep diary every day
BI3	I will definitely do the relaxation exercise every day
BI4	I will certainly stay compliant to the bedtimes that I have agreed with the coach
BI5	I am going to follow up on the sleep tips
BI6	I am going to fill in my sleep times in the sleep diary as well as possible
BI7	I am going to take the time to do the relaxation exercise every day

### Facilitating conditions

**Table:**

FC1	I have enough knowledge necessary for using the SleepCare app
FC2	My mobile phone works perfectly for running the SleepCare app
FC3	Where necessary, the SleepCare team will help me to use the SleepCare app
FC4	Where necessary, my friends and/or family will help me to use the SleepCare app

## Appendix B data, analysis, and results of the multiple-path analysis

### Description analysis of preprocessed data

The following results were taken before the imputation procedure (n = 89).

**Table:**

Items		NA	M	SD	Range
Behavioral Intention					
	BI1		5.88	1.46	[1.0.7]
	BI2		5.88	1.51	[1.0.7]
	BI3		3.69	1.87	[1.0.7]
	BI4	8	4.83	1.71	[1.0.7]
	BI5	2	5.79	1.25	[1.0.7]
	BI6	3	6.35	1.18	[1.0.7]
	BI7		4.40	1.93	[1.0.7]
Performance Expectancy					
	PE1	1	4.78	1.70	[1.0.7]
	PE2	2	4.21	1.81	[1.0.7]
	PE3	1	4.45	1.55	[1.0.7]
	PE4	1	5.88	1.36	[1.0.7]
	PE5	3	4.30	1.67	[1.0.7]
	PE6	6	5.67	1.62	[1.0.7]
	PE7	2	4.31	1.67	[1.0.7]
Effort Expectancy					
	EE1	1	5.40	1.56	[1.0.7]
	EE2	1	5.95	1.22	[1.0.7]
	EE3	1	6.31	1.14	[1.0.7]
	EE4	1	6.24	1.16	[1.0.7]
	EE5-R	1	6.12	1.45	[1.0.7]
	EE6	1	5.69	1.31	[1.0.7]
Social Influence					
	SI1	4	4.94	1.45	[1.0.7]
	SI2	4	5.02	1.57	[1.0.7]
	SI3	11	4.58	1.58	[1.0.7]
	SI4	5	5.19	1.44	[1.0.7]
	SI5-R	4	6.21	1.26	[1.0.7]
Hedonic Motivation					
	HM1	2	5.77	1.34	[1.0.7]
	HM2-R	2	6.03	1.21	[1.0.7]
	HM3	2	4.89	1.54	[1.0.7]
	HM4	2	5.41	1.39	[1.0.7]
	HM5	3	4.81	1.70	[1.0.7]
	HM6	10	4.46	1.69	[1.0.7]
	HM7-R	4	6.22	1.34	[1.0.7]
Self-Efficacy					
	SE1	2	6.69	0.83	[1.0.7]
	SE2	14	5.23	2.10	[1.0.7]
	SE3	2	6.78	0.77	[1.0.7]
	SE4	2	6.13	1.32	[1.0.7]
Trust					
	TR1	2	5.63	1.17	[1.0.7]
	TR2-R	2	5.14	1.80	[1.0.7]
	TR3	2	5.63	1.36	[1.0.7]
Anxiety					
	AN1-R	2	6.52	1.16	[1.0.7]
	AN2-R	2	6.21	1.44	[1.0.7]
	AN3-R	2	6.05	1.75	[1.0.7]
	AN4-R	2	6.51	1.14	[1.0.7]
Facilitating Conditions					
	FC1	3	6.31	1.35	[1.0.7]
	FC2	2	6.45	1.16	[1.0.7]
	FC3	40	5.20	1.90	[1.0.7]
	FC4	55	3.32	2.06	[1.0.7]
Use-Behavior					
	SD-DO	2	34.49	13.42	[0.0.1]
	CONV-DO	2	13.05	5.42	[0.0.1]
	RE-DO	2	13.86	12.01	[0.0.1]

*NA* missing cases, *M* Mean, *SD* Standard Deviation

### Summary of variability and assessment results

The following results were taken after the imputation procedure (n = 89).

**Table:**

Construct	Indicators	Variability					Assessment Result	
		**Mean**	**Variance**	**SD**	**Range**	**IQR**	**Inclusion**	**Exclusion Criteria**
Performance Expectancy	PE1	4.74	3.01	1.74	[1.0.7]	2	✔	
	PE2	4.25	3.28	1.81	[1.0.7]	3	✔	
	PE3	4.42	2.52	1.49	[1.0.7]	2	✔	
	PE4	5.82	2.10	1.45	[1.0.7]	2	✔	
	PE5	4.21	2.94	1.72	[1.0.7]	3	✔	
	PE6	5.64	2.92	1.71	[1.0.7]	2	✔	
	PE7	4.30	2.94	1.71	[1.0.7]	3	✔	
Effort Expectancy	EE1	5.42	2.45	1.57	[1.0.7]	2	✔	
	EE2	5.92	1.57	1.57	[1.0.7]	1	✔	
	EE3	6.31	1.29	1.13	[1.0.7]	1	✔	
	EE4	6.20	1.47	1.21	[1.0.7]	1	✔	
	EE5-R	6.12	2.09	1.44	[1.0.7]	1	✘	Loading < 0.50
	EE6	5.66	1.77	1.33	[1.0.7]	2	✔	
Social Influence	SI1	4.94	2.19	1.48	[1.0.7]	2	✔	
	SI2	4.92	2.85	1.69	[1.0.7]	2	✔	
	SI3	4.63	2.67	1.63	[1.0.7]	2	✘	NA > Q3 + 1.5*IQRq
	SI4	5.18	2.31	1.52	[1.0.7]	2	✔	
	SI5-R	6.16	1.79	1.34	[1.0.7]	1	✘	Loading < 0.50
Self-Efficacy	SE1	6.67	0.70	0.84	[1.0.7]	0	✔	
	SE2	5.10	5.18	2.28	[1.0.7]	3	✘	NA > Q3 + 1.5*IQRq
	SE3	6.79	0.59	0.76	[1.0.7]	0	✔	
	SE4	6.03	2.08	1.44	[1.0.7]	1	✔	
Trust	TR1	5.66	1.39	5.66	[1.0.7]	1	✔	
	TR2-R	5.18	3.24	1.39	[1.0.7]	3	✘	AVE < 0.50
	TR3	5.66	1.84	1.36	[1.0.7]	2	✔	
Hedonic Motivation	HM1	5.73	2.04	1.43	[1.0.7]	2	✔	
	HM2-R	6.06	1.46	1.21	[2.0.7]	1	✔	
	HM3	4.93	2.40	1.55	[1.0.7]	2	✔	
	HM4	5.45	1.95	1.40	[1.0.7]	2	✔	
	HM5	4.82	3.06	1.75	[1.0.7]	2	✔	
	HM6	4.94	3.32	1.82	[1.0.7]	2	✘	NA > Q3 + 1.5*IQRq
	HM7-R	6.07	2.52	1.59	[1.0.7]	1	✘	Loading < 0.50
Anxiety	AN1-R	6.53	1.32	1.15	[1.0.7]	0	✔	
	AN2-R	6.22	2.04	1.43	[2.0.7]	1	✘	Loading < 0.50
	AN3-R	6.00	3.30	1.82	[1.0.7]	1	✘	Loading < 0.50
	AN4-R	6.52	1.28	1.13	[1.0.7]	0	✔	
Facilitating Conditions	FC1	5.25	2.01	1.42	[1.0.7]	1	✔	
	FC2	6.44	1.34	1.16	[1.0.7]	1	✔	
	FC3	6.97	4.74	2.18	[1.0.7]	3	✘	NA > Q3 + 1.5*IQRq
	FC4	3.25	3.85	1.96	[1.0.7]	3	✘	NA > Q3 + 1.5*IQRq
Behavioral Intention	BI1	5.88	2.13	1.46	[1.0.7]	1	✔	
	BI2	5.88	2.29	1.51	[1.0.7]	1	✔	
	BI3	3.69	3.51	1.87	[1.0.7]	3	✔	
	BI4	4.84	3.29	1.81	[1.0.7]	2	✘	NA > Q3 + 1.5*IQRq
	BI5	5.81	1.54	1.24	[1.0.7]	2	✔	
	BI6	6.22	1.79	1.34	[1.0.7]	1	✔	
	BI7	4.40	3.72	1.93	[1.0.7]	3	✔	
Use-Behavior	SD-DO	34.65	172.80	13.15	[0.0.49]	14	✔	
	CONV-DO	12.78	31.93	5.65	[0.0.25]	8	✔	
	RE-DO	13.89	146.08	12.09	[0.0.45]	17	✔	

*SD* Standard Deviation, *NA* Missing Data, *IQR* Interquartile range, *IQRq* Interquartile range of the ‘not applicable’ frequency across the questionnaire item measured

### PLS algorithm results of the multiple-path analysis

The following results were based on the multiple-path test.

**Table:**

Construct	Indicator	Loading > 0.50	Cronbach’s α > 0.70	AVE > 0.50	Discriminant Validity
Performance Expectancy			0.87	0.55	Yes
	PE1	0.76			
	PE2	0.67			
	PE3	0.88			
	PE4	0.68			
	PE5	0.81			
	PE6	0.67			
	PE7	0.70			
Effort Expectancy			0.90	0.72	Yes
	EE1	0.74			
	EE2	0.89			
	EE3	0.88			
	EE4	0.89			
	EE6	0.82			
Social Influence			0.83	0.75	Yes
	SI1	0.90			
	SI2	0.91			
	SI4	0.79			
Self-Efficacy			0.84	0.76	Yes
	SE1	0.90			
	SE3	0.91			
	SE4	0.79			
Trust			0.76	0.81	Yes
	TR1	0.91			
	TR3	0.89			
Hedonic Motivation			0.81	0.57	Yes
	HM1	0.80			
	HM2-R	0.71			
	HM3	0.86			
	HM4	0.78			
	HM5	0.60			
Anxiety			0.76	0.80	Yes
	AN1-R	0.94			
	AN4-R	0.85			
Facilitating Condition			0.66	0.74	Yes
	FC1	0.91			
	FC2	0.81			
Behavioral Intention			0.86	0.58	Yes
	BI1	0.87			
	BI2	0.87			
	BI3	0.67			
	BI5	0.71			
	BI6	0.77			
	BI7	0.68			
Use-Behavior			0.82	0.74	Yes
	CONV-DO	0.85			
	SD-DO	0.90			
	RE-DO	0.82			

### Bootstrapping results of the multiple-path analysis (Fig. 3)

**Table:**

					Predictor				
	**Construct**		***Q*****2**	***β***	**95% CI**				
*H*		***R***^**2**^			**Low**	**High**	**Bias**	***f***^**2**^	***q***^**2**^
Behavioral Intention as a dependent variable		0.61	0.30						
H1	Performance Expectation			0.09	–0.17	0.27	0.04	0.01	
H2	Effort Expectation			0.19	–0.03	0.44	–0.01	0.03	
H3	Social Influence			0.05	–0.15	0.24	0.01	0.00	
H4	Self-Efficacy			0.04	–0.26	0.27	0.02	0.00	
H5	Trust			0.15	–0.09	0.43	–0.03	0.03	
H6	Hedonic Motivation			0.32*	0.10	0.55	0.00	0.12	0.03
H7	Anxiety			0.15	–0.12	0.42	0.00	0.05	
H8	Facilitating Conditions			0.17	–0.05	0.46	–0.03	0.04	
Use-Behavior as a dependent variable		0.32	0.21						
H9	Behavioral Intention			0.41*	0.16	0.60	0.02	0.18	0.09
H10	Facilitating Conditions			0.25*	0.003	0.46	0.00	0.07	0.03

Note: *significant, i.e. zero outside 95% confidence interval

### Bootstrapping results of the mediation analysis (Fig. 4)

**Table:**

Independent Variable (IV)	Dependent Variable (DV)	Mediator (M)	*n*	Effect		95% CI		Bias
						**Low**	**High**	
Hedonic Motivation	Use-Behavior	Behavioral Intention	89	*βc* =	0.42*	0.21	0.55	0.02
				*βa* =	0.67*	0.51	0.76	0.02
				*βb* =	0.44*	0.15	0.66	0.00
				*βaxb* =	0.29*	0.09	0.46	0.01
				*βc’* =	0.13	–0.13	0.36	0.01

*βa* is an effect of IV on M, *βb* is an effect of M on DV, *βc* is a direct effect of IV on DV without mediation, *βaxb* is an indirect effect of IV on DV (*βa* x *βb*), *βc*’ is a direct effect of  IV on DV with mediation (*βc* - *βaxb*), *IV* is Independent Variable, *M* is Mediation Variable, *DV* is Dependent Variable

*significant, i.e. zero outside 95% confidence interval
